# KANPM-DTA: improving drug–target affinity prediction with Kolmogorov–Arnold networks and pretrained models

**DOI:** 10.1093/bib/bbag112

**Published:** 2026-03-12

**Authors:** M D Youshuf Khan Rakib, Muhammad Habibulla Alamin, Jiamu Li, Sheikh Sohan Mamun, Kaleb Amsalu Gobena, Shengbing Ren

**Affiliations:** School of Computer Science and Engineering, Central South University, Changsha, 410083 Hunan, China; School of Computer Science and Engineering, Central South University, Changsha, 410083 Hunan, China; School of Computer Science and Engineering, Macau University of Science and Technology, Taipa, 999078 Macau, China; School of Computer Science and Engineering, Central South University, Changsha, 410083 Hunan, China; School of Computer Science and Engineering, Central South University, Changsha, 410083 Hunan, China; School of Computer Science and Engineering, Central South University, Changsha, 410083 Hunan, China

**Keywords:** drug–target affinity, graph neural networks, attention mechanisms, pretrained models, Kolmogorov–Arnold networks

## Abstract

Accurate drug–target affinity (DTA) prediction is critical for drug discovery and repurposing. However, existing models often struggle with generalizing to unseen drug–target pairs, lack interpretability, and fail to integrate heterogeneous biological features effectively. To overcome these challenges, we introduce KANPM-DTA, a deep learning framework designed to capture richer biochemical interactions and improve prediction reliability. Specifically, an ESM-guided protein graph construction strategy incorporates evolutionary and structural information to overcome underexplored protein representations. A gated fusion mechanism was employed to integrate drug–protein graph features, while linear attention captures cross-modal dependencies that enhance discriminative power. For the final affinity prediction, a Kolmogorov–Arnold network was used, offering a stronger nonlinear approximation and improved interpretability. Comprehensive experiments on benchmark datasets demonstrate that KANPM-DTA significantly outperforms state-of-the-art methods. On the Davis, KIBA, Metz, and BindingDB datasets, we achieved significant performance improvements under warm setting, with MSE reductions of 6.42%, 4.86%, 4.44%, and 5.46%, CI increases of 0.45%, 0.34%, 0.48%, and 0.80%, and $r_{m}^{2}$ gains of 1.85%, 0.90%, 0.84%, and 1.05%, respectively. Moreover, a case study on the epidermal growth factor receptor further highlights the effectiveness of KANPM-DTA in predicting DTAs for unknown drug–target pairs, emphasizing its potential for real-world applications in drug discovery. However, wet-lab validation is required to assess the applicability of the results.

## Introduction

Predicting drug–target affinity (DTA) is a critical task in computational drug discovery, providing insights into the interactions between small molecules and proteins. Traditional experimental methods, such as molecular docking and affinity assays [1], are resource-intensive but costly and cannot be applied to large-scale exploration of chemical and biological spaces. Therefore, deep learning-based models offer scalable, cost-effective alternatives by directly learning complex biochemical interaction patterns from small molecule of drugs and protein characteristics.

Based on the representation of input data, these deep learning-based affinity prediction methods can be classified into two categories: sequence-based methods and graph-based methods [2]. Sequence-based methods mainly represent drugs and proteins as 1D sequences and extract global features of proteins and drugs through separate encoders [3]. However, these methods fail to capture local features from protein–ligand inputs, which may result in the loss of crucial information and reduced prediction accuracy.

In contrast to sequence-based methods, graph-based methods focus more on the structural information on drugs and targets, and while they offer more detailed representations, challenges remain in modeling cross-modal interactions effectively. For instance, GraphDTA [4] introduced graph neural networks (GNNs) for drug encoding, but it employed a simple CNN for protein encoding, limiting its ability to generalize across different protein families. FusionDTA [5] and MgraphDTA [6] explored multimodal fusion strategies by combining molecular graphs with protein sequences, but they relied on simple concatenation, neglecting the complex interactions between modalities.

Recent advancements, such as MSGNN-DTA [7], have incorporated message-passing GNNs and mutual attention between drug and protein features. However, the lack of interaction modeling and overreliance on pairwise matching limit their generalization ability, especially under cold-start conditions. Similarly, GRA-DTA [8] introduced relation-aware attention to enhance the interaction modeling, but its reliance on handcrafted priors restricted adaptability to unseen drug–target pairs.

To improve generalizability, MMSG-DTA [9] introduced a multiscale and multimodal fusion network, though it required extensive hyperparameter tuning and lacked interpretability. Similarly, PMMR [10] used pretrained molecular and protein representations but treated local and global features as separate streams, lacking unified attention. DMFF-DTA [11] combines protein and drug sequence embeddings with protein and drug molecular graphs. However, it uses standard LSTM models for sequence encoding and shallow graph encoders, without leveraging pretrained models, which limits its ability to capture rich, context-aware features and reduces generalization, especially for novel drug–target pairs.

To overcome these challenges, we present a novel model named KANPM-DTA, that leverages pretrained molecular and protein language models to construct rich multi-view representations. Specifically, ESM-Cambrian (ESM-C) [12] and ChemBERTa-2 [13] were used to extract high-dimensional sequence embeddings for proteins and drugs, respectively. In addition, KANPM-DTA introduces a novel protein graph construction strategy using contact maps predicted by ESM-2 [14] to define the edge structures, with node features derived from ESM-C residue-level embeddings, inspired by SP-DTI [15] and FlexMol [16]. To the best of our knowledge, this is the first application of an ESM-C guided graph formulation in DTA prediction, offering deeper insights into protein interactions. The model also incorporates a gated fusion module that combines protein and drug graph features, capturing both intra- and inter-sequence dependencies through linear attention mechanisms. For the final affinity prediction, Kolmogorov–Arnold networks (KAN) [17] were employed instead of traditional MLPs [18], enhancing the models expressiveness and interpretability. Comprehensive experiments conducted on benchmark datasets demonstrate that KANPM-DTA outperforms existing models in terms of accuracy and generalizability. A case study focusing on lung cancer targeted protein further highlights the models real-world applicability, including its potential for drug repurposing.

## Materials and methods

### Datasets

To evaluate our model, we carried out experiments on four widely used benchmark datasets: Davis [19], KIBA [20], Metz [21], and BindingDB [22]. These datasets cover diverse drug–target interaction(DTI) scenarios and represent standard testbeds for binding affinity prediction. The Davis dataset focuses on the binding affinity of 68 kinase inhibitors against 442 kinases, providing a total of 30 057 interaction pairs. The affinity values are measured in terms of the dissociation constant $K_{d}$ and are transformed to the logarithmic scale $pK_{d}$ to ensure numerical stability. The KIBA dataset integrates multiple bioactivity measures (including $K_{i}$, $K_{d}$, and IC$_{50}$) into a unified KIBA score. It consists of 118 083 interaction pairs involving 2111 drugs and 228 target proteins, offering a broad chemical space with rich biological diversity. The Metz dataset serves as an additional benchmark to test the model’s robustness. It includes 35 259 binding interactions across 1423 compounds and 170 target proteins, with affinity scores derived from publicly available assay results. The BindingDB dataset is a widely used resource containing experimentally determined binding affinities between proteins and small-molecule ligands. For this study, we utilized a curated subset consisting of 1620 targets and 18 044 drugs, which includes various affinity measures such as dissociation constants (Kd), inhibition constants (Ki), and IC50 values. For all datasets, we follow standard practice by splitting the interactions into training, validation, and test sets in an 8:1:1 ratio, ensuring non-overlapping drug–target pairs to assess generalization under cold-start conditions. Table 1 summarizes the key statistics of the four datasets.

**Table 1:** Details of benchmark datasets.

Datasets	Drugs	Proteins	Training	Validation	Test
Davis	68	442	24 045	3006	3006
Kiba	2111	228	94 467	11 808	11 808
Metz	1423	170	28 207	3526	3526
BindingDB	18 044	1620	45 220	5653	5653

### Model architecture

**Figure 1:**
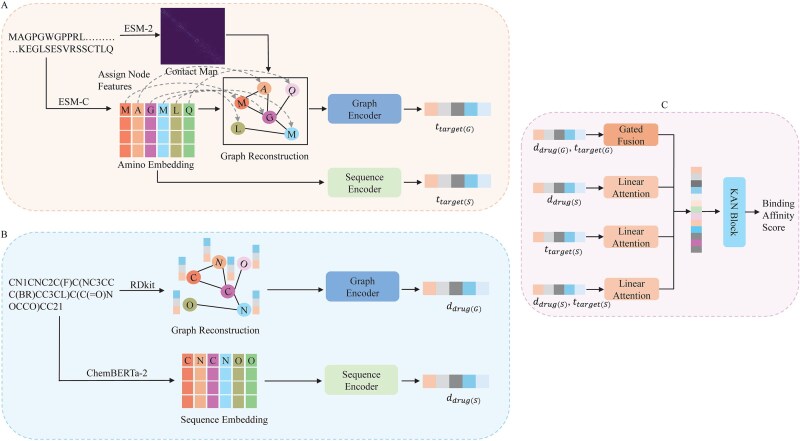
The proposed KANPM-DTA model architecture. (A) Graph and sequence-based protein feature encoding. (B) Graph and sequence-based drug feature encoding. (C) Adaptive feature learning between drug and protein graph-sequence features.

The overall architecture of KANPM-DTA is illustrated in Figure 1. It consists of two primary encoding modules: one for protein and the other for drug. For protein encoding, a protein graph is constructed using contact maps predicted by ESM-2, with node features derived from ESM-C embeddings. This protein graph is then passed through a stacked GCN-GAT-based graph encoder to extract graph-level features. Simultaneously, ESM-C protein sequence embeddings are processed through a Transformer-based sequence encoder to capture sequential features. Similarly, For drug encoding drug graphs are constructed using RDKit [23] and encoded via a graph encoder, while SMILES-based embeddings from ChemBERTa-2 are fine-tuned using a sequence encoder to capture sequential drug features [24]. These four components protein–drug graph and sequence features are processed concurrently to obtain comprehensive protein and drug representations. In the adaptive learning module, graph features are fused using a gated fusion mechanism, and linear attention is applied to the sequence features and their combinations. Finally, all representations are fed into a KAN block to predict the binding affinity.

#### Protein sequence encoding

Each target protein is described by its amino acid sequence in the standard FASTA format, which is then encoded using ESM-C (600 M), a large-scale transformer-based protein language model pretrained on over 2.7 billion unique protein sequences. ESM-C is optimized to capture biologically relevant protein representations and excels at representation learning, leveraging pretrained embeddings from large and diverse protein datasets. The ESM-C embeddings significantly contribute to the model’s generalization capabilities by capturing generalizable protein features, including evolutionary and functional information. This enables the model to recognize shared patterns across proteins. Unlike the ESM-3 generative model, which focuses on controllable sequence generation, ESM-C excels in handling multi-strand inputs to model complex protein interactions without requiring structural data [25]. The model’s ability to process a wide range of protein sequences enhances its robustness, allowing it to transfer sequence-level information across different biological contexts. This transferability enables ESM-C to generalize and predict complex protein interactions effectively, further strengthening its predictive accuracy in real-world scenarios. The initial features extracted by ESM-C are represented in Equation (1):


(1) \begin{align*}& E_{t} = \mathrm{ESM-C}(X_{t}) W_{x} \in \mathbb{R}^{n \times v_{t}}\end{align*}

where $X_{t}$ is the input sequence, the raw amino acid sequence of the protein, $n$ is the length of the sequence, $v_{t}$ is the dimension of the hidden layer in the pretraining model, and $W_{x}$ is a trainable weight matrix.

To adapt the pretrained features to downstream tasks, we use a transformer to fine-tune the pretrained features. At the same time, due to the inconsistency in protein sequence lengths, we take the largest sequence length in each batch as the unified length. The resulting features are expressed in Equation (2):


(2) \begin{align*}& F_{t} = \mathrm{Transformer}(E_{t})\end{align*}

#### Protein graph encoding

In parallel to sequence encoding, we build a weighted residue-level graph to model the structural dependencies within each protein. To do this, we employ the ESM-2 (3B) model to generate a contact map, which predicts the spatial proximity of amino acid residues in pairs within the 3D fold of the protein. Specifically, given a protein sequence $X_{t}$, ESM-2 outputs a contact probability matrix:


(3) \begin{align*}& M_{ij} = f_{\mathrm{ESM2}}(r_{i}, r_{j}), \quad E_{p} = \{(i,j) \mid M_{ij}> 0.5\}, \quad W_{ij} = M_{i,j}\end{align*}

where $M_{i,j}$ is the contact probability between residues $r_{i}$ and $r_{j}$, $E_{p}$ is the set of edges in the protein graph, formed if the contact probability exceeds a threshold of 0.5, $W_{i,j}$ is the edge weight corresponding to the contact probability between residues $r_{i}$ and $r_{j}$ [26].

Meanwhile, the node features for each residue are directly taken from the sequence embeddings generated by the ESM-C model as described in Section Protein sequence encoding. After obtaining the full sequence embedding matrix $E_{t} \in \mathbb{R}^{n \times v_{t}}$ with hidden size $v_{t} = 1152$, we discard the special start and end tokens to match the number of residue tokens with the graph nodes, ensuring alignment between the sequence representation and the residue graph topology. This yields residue node features:


(4) \begin{align*}& V_{p} = \{v_{i} = s_{t_{i}}\} \quad \mathrm{for} \quad i = 1,2,3,\ldots,n-2\end{align*}

where $V_{p}$ is the set of residue node features, $v_{i}$ is the feature vector for each residue $i$, taken directly from the sequence embeddings $s_{t_{i}}$.

The final weighted protein graph is thus constructed as $G_{p} = (V_{p}, E_{p}, W_{p})$. To maintain computational consistency during mini-batch training, all protein graphs are padded to match the maximum residue length in each batch.

**Figure 2:**
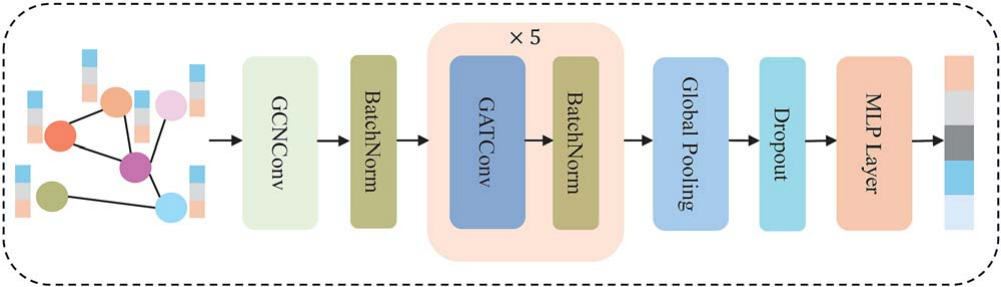
The architecture of graph encoder.

Once constructed, each protein graph is passed through a dedicated multi-layer graph encoder illustrated in Figure 2 to extract robust structural representations. First, a GCNConv layer aggregates local context based on the weighted adjacent matrix derived from the contact map:


(5) \begin{align*}& H_{p}^{1} = \mathrm{GCNConv}(V_{p}, E_{p}, W_{p}), \quad \widetilde{H}_{p}^{1} = \mathrm{BN}(H_{p}^{1})\end{align*}

Next, five stacked GATConv blocks refine the node embeddings by applying learned residue-level attention, capturing diverse intra-protein substructures:


(6) \begin{align*} & H_{p}^{(l+1)} = \mathrm{GATConv}(\widetilde{H}_{p}^{l}, E_{p}) \end{align*}

(7) \begin{align*} & \quad \widetilde{H}_{p}^{(l+1)} = \mathrm{BN}(H_{p}^{(l+1)}), \quad l=1,\ldots,5. \end{align*}

Finally, a global add pooling operation aggregates all refined residue embeddings into a single graph-level protein representation:


(8) \begin{align*}& H_{p} = \mathrm{MLP}(\mathrm{Dropout}(\mathrm{GlobalAddPool}(\widetilde{H}_{p}^{6})))\end{align*}

Through this combined sequence-to-graph construction, ESM-2 contact map, and ESM-C residue embeddings, our model integrates both evolutionary sequence context and spatial residue relationships, yielding a protein representation, i.e. highly informative for downstream drug–target binding prediction.

#### Drug sequence encoding

Each drug candidate is represented by its canonical SMILES string, which encodes its atom sequence and connectivity in text form. To obtain the pretrained features of drug SMILES strings, we use the chemical language model ChemBERTa-2 (masked language modeling, MLM). ChemBERTa-2 is a BERT-like transformer model that learns molecular fingerprints through semi-supervised pretraining of the language model. ChemBERTa-2 employs MLM and multi-task regression over a large corpus of 77 million SMILES strings, a well-known text representation of molecules.


(9) \begin{align*}& E_{s} = \mathrm{ChemBERTa}(X_{s}) W_{x} \in \mathbb{R}^{n \times v_{s}}\end{align*}

where $X_{s}$ is the input SMILES string for the drug, $n$ is the sequence length of the drug SMILES string, $v_{s}$ is the dimension of the hidden layer in the pretrained model for drug sequences.

In order to ensure the length consistency of SMILES in each batch, we also take the maximum SMILES length as the unified length. Then, a transformer is used to fine-tune pretrained SMILES features:


(10) \begin{align*}& F_{s} = \mathrm{Transformer}(E_{s})\end{align*}

#### Drug graph encoding

The SMILES string of each drug molecule is first parsed using RDKit, which generates a weighted molecular graph $G_{d} = (V_{d}, E_{d}, W_{d})$. Each node $v_{k} \in V_{d}$ represents an atom and is described by an 88D feature vector summarizing its elemental type, charge, hybridization, hydrogen count, aromaticity, and related properties. The complete list of node features used in our model is detailed in Table S1 in Supplementary File 1. Each edge $(k,l) \in E_{d}$ denotes a chemical bond, and its weight $w_{kl}$ equals the bond order, ensuring the graph naturally encodes chemical strength information. To ensure computational consistency when training on multiple molecules of varying sizes, we pad each molecular graph to match the maximum number of nodes within each batch, enabling uniform batched processing throughout the model.

After construction and batching, each drug graph is passed through a multi-layer graph encoder to extract meaningful structural representations. Specifically, the graph first passes through a GCNConv layer that aggregates information from neighboring nodes considering the weighted adjacency matrix:


(11) \begin{align*}& H_{d}^{1} = \mathrm{GCNConv}(V_{d}, E_{d}, W_{d}), \quad \widetilde{H}_{d}^{1} = \mathrm{BN}(H_{d}^{1})\end{align*}

Next, five stacked GATConv blocks refine this representation by applying learned attention to neighbors, capturing diverse substructures, and flexible dependencies:


(12) \begin{align*} & H_{d}^{(l+1)} = \mathrm{GATConv}(\widetilde{H}_{d}^{l}, E_{d}) \end{align*}

(13) \begin{align*} & \quad \widetilde{H}_{d}^{(l+1)} = \mathrm{BN}(H_{d}^{(l+1)}), \quad l=1,\ldots,5. \end{align*}

Finally, a global add pooling operation aggregates all atom-wise embeddings into a single graph-level drug feature vector:


(14) \begin{align*}& H_{d} = \mathrm{MLP}(\mathrm{Dropout}(\mathrm{GlobalAddPool}(\widetilde{H}_{d}^{6})))\end{align*}

The resulting $H_{d}$ provides a robust representation of the drug’s molecular structure, enriched with both local bonding details and global topological context.

#### Interaction module

After obtaining the comprehensive feature representations for both drug and target protein, an adaptive feature extraction module is employed to effectively capture the complex inter-dependencies between these heterogeneous modalities. The aim is to bridge local structural features learned from the multi-layer GCN-GAT [27, 28] graph encoders with global sequential patterns extracted by the transformers, enabling the model to reason jointly over chemical structure and bio-sequence context.

First, the graph-level embeddings $H_{d}$ and $H_{p}$ are passed through a gated fusion layer:


(15) \begin{align*}& A_{g} = \sigma(W_{g} [H_{d}; H_{p}]) \odot H_{d} + (1 - \sigma(W_{g} [H_{d}; H_{p}])) \odot H_{p}\end{align*}

where $[H_{d};\, H_{p}]$ denotes concatenation, $W_{g}$ is a learnable parameter, and $\sigma $ denotes the sigmoid activation.

Meanwhile, The fine-tuned sequence representations $F_{s}$ and $F_{t}$ are each passed through a linear attention module to distill the most informative sequence-level cues:


(16) \begin{align*} & A_{d} = \mathrm{LinearAttn}(F_{s}) \end{align*}

(17) \begin{align*} & A_{t} = \mathrm{LinearAttn}(F_{t}) \end{align*}

The outputs are then concatenated and processed by an additional linear attention block to explicitly model cross-modality dependencies:


(18) \begin{align*}& A_{dt} = \mathrm{LinearAttn}([A_{d}, A_{t}])\end{align*}

Next, the model aggregates these diverse signals by concatenating the fused graph-level representation with the refined sequence-level attentions:


(19) \begin{align*}& Z = {\mathrm{Concat}}([A_{g}, A_{d}, A_{t}, A_{dt}])\end{align*}

#### Prediction module

Finally, the concatenated multi-source representation $ Z $ is fed into KAN block, which serves as a powerful regression head to predict the drug–target binding affinity. The regression task is formulated as follows:


(20) \begin{align*}& \hat{y} = \text{KAN Block}(Z)\end{align*}

where $ \hat{y} $ is the predicted binding affinity. This prediction is made based on the learned interactions between drug and protein features, with the KAN enabling more accurate and interpretable results. The detailed architecture of the KAN block is provided in Section Details of KAN Block, Table S2, and Fig. S1 in Supplementary File 1.

## Experiments and results

### Evaluation strategies

In this study, the experiments were implemented under the following settings:

- Warm setting: The dataset is randomly partitioned, allowing drug and protein identifiers in the test set can also appear in the training and validation sets.
- Unseen-drug (cold-start): The dataset is partitioned according to distinct drug identifiers, ensuring that all drugs in the test set are completely absent from the training and validation sets.
- Unseen-protein (cold-start): The dataset is partitioned according to distinct protein identifiers so that all proteins in the test set are completely absent from the training and validation sets.
- All-unseen (cold-start): The dataset is jointly split based on drug and protein identifiers, ensuring that both drugs and proteins in the test set are completely unseen during training and validation, thus representing the most stringent cold-start scenario.

### Evaluation metrics

We evaluate the performance of the proposed KANPM-DTA model using three standard regression metrics on benchmark DTA datasets: mean squared error (MSE), Concordance Index (CI) [29], and the mean reversion coefficient ($r_{m}^{2}$) [30]. MSE measures the average squared difference between predicted and actual binding affinity values. A smaller MSE indicates that the predicted values are closer to the true values. The MSE defined in Equation (21):


(21) \begin{align*}& \mathrm{MSE} = \frac{1}{N} \sum_{i=1}^{N} (y_{i} - p_{i})^{2}\end{align*}

where $y_{i}$ represents the true value of the $i$th sample, and $p_{i}$ is the predicted value of the $i$th sample.

CI evaluates the ability of the model to correctly rank pairs of DTIs based on their predicted affinities. A higher CI indicates better predictive performance of the model:


(22) \begin{align*}& \mathrm{CI} = \frac{1}{Z} \sum_{y_{i}> y_{j}} h(p_{i} - p_{j})\end{align*}

where $p_{i}$ is the predicted value corresponding to the true affinity value $y_{i}$, and $p_{j}$ is the predicted value corresponding to the true affinity value $y_{j}$. $h(x)$ is the step function, and $Z$ is the normalization constant that maps the values to the range [0,1]:


(23) \begin{align*}& h(x) = \begin{cases} 1, & x> 0 \\ 0.5, & x = 0 \\ 0, & x < 0 \end{cases}\end{align*}

The $r_{m}^{2}$ metric is used to assess the external predictive reliability of the model and its tendency to regress toward the mean. A model with a higher $r_{m}^{2}$ value for the test set is considered acceptable:


(24) \begin{align*}& r_{m}^{2} = r^{2} \times \left(1 - \sqrt{r^{2} - r_{0}^{2}}\right)\end{align*}

where $r$ is the correlation coefficient with an intercept, and $r_{0}$ is the correlation coefficient without an intercept.

### Experimental settings

We utilized PyTorch framework to implement the proposed KANPM-DTA model. The training process was executed on an NVIDIA Tesla V100-PCIE GPU with 32 GB of memory. We employed the Adam optimizer with an initial learning rate of 0.0001, and set the maximum number of training epochs to 200. Early stopping was applied if the MSE value on the validation set did not decrease for >20 consecutive epochs. The complete set of hyperparameters and model architecture specifications are provided in Table 2. Experiments were conducted on the Davis, KIBA, Metz, and BindingDB datasets described in Section Datasets, using the evaluation metrics MSE, CI, and $r_{m}^{2}$ introduced in Section Evaluation metrics. Each experiment was repeated $N=5$ times with different random seeds, and the mean and standard deviation of these results were reported. The details of molecular docking studies are provided in Table S3 in Supplementary File 1.

**Table 2:** Hyperparameter settings of KANPM-DTA.

Hyperparameter	Value
Epoch	200
Learning rate	0.0001
Batch size	16
Dropout rate	0.2
Optimizer	Adam
Transformer encoder layers	3
Transformer encoder heads	8
Transformer encoder output dim	128
Transformer encoder hidden dim	128
Protein sequence maximum length	1200
Protein graph encoder input dim	1152
Protein graph encoder output dim	128
Protein graph encoder hidden dim	256
Drug sequence maximum length	220
Drug graph encoder input dim	384
Drug graph encoder output dim	128
Drug graph encoder hidden dim	128

### Baseline models

To comprehensively evaluate KANPM-DTA, we compare it against the following representative DTA models:

- DeepDTA [3] encodes drug SMILES and protein sequences with separate 1D CNNs and concatenates the latent vectors for MLP-based affinity regression.
- GraphDTA [4] represents drugs as molecular graphs with GNN encoders, encodes proteins via 1D CNNs, and concatenates both embeddings for prediction.
- FusionDTA [5] uses lightweight sequence encoders for drugs and proteins and an attention-based feature polymerizer to fuse multi-level representations before MLP regression.
- MGraphDTA [6] applies a deep multiscale GNN with dense connections to drug graphs, combines the embeddings with CNN-based protein features, and predicts affinity via fully connected layers.
- MSGNN-DTA [7] extracts multi-scale topological features for drug and protein graphs using GNNs and fuses them with gated skip-connections before a regression head.
- GPCNDTA [31] employs GNNs and self-attention to encode drug graphs and protein sequences, then uses intra- and inter-molecular cross-attention to fuse features for affinity prediction.
- GRA-DTA [8] encodes proteins with BiGRU plus soft attention, drugs with GraphSAGE, and applies an attention-based fusion module before dense layers.
- LLMDTA [32] leverages Mol2Vec and ESM2 pretrained embeddings for drugs and proteins, adapts them with 1D CNNs, and used bi-linear attention-based interaction the vectors for MLP-based regression.
- TDGraphDTA [33] builds multi-scale drug graphs, learns extended graph representations of molecular substructures, and combines them with protein features for dense-layer prediction.
- MDCT-DTA [33] introduces a multi-scale diffusion/graph convolution module on drug graphs, fuses multi-scale drug and protein features, and outputs affinity via dense layers.
- PMMR [34] learns multi-view representations from pretrained SMILES and graph encoders and applies prediction risk minimization and fusion layers to enhance generalization.
- DMFF-DTA [11] jointly models sequence and graph information for drugs and proteins with binding-site-focused protein graphs and fusion blocks before affinity regression.
- MMSG-DTA [9] integrates multiscale GNNs and sequence encoders on both drug and protein modalities, fusing multiscale features, and using dense layers for DTA prediction.
- MMFA-DTA [35] is a multimodal DTA model that jointly encodes drug and protein graphs and sequences at multiple granularities, and uses cross-modal attention fusion to integrate these heterogeneous features before affinity regression.

### Performance comparisons with baseline models on warm settings

To comprehensively evaluate the predictive performance of our proposed KANPM-DTA, we conducted comparative experiments against a diverse range of classic and state-of-the-art (SOTA) DTA prediction methods. These include sequence-based models such as DeepDTA [3], FusionDTA [5], and graph-based architectures such as GraphDTA [4], MgraphDTA [6], MSGNN-DTA [7], GRA-DTA [8], TDGraphDTA [33], as well as hybrid approaches like GPCNDTA [31], LLMDTA [32], MDCT-DTA [34], MMFA-DTA [35], PMMR [10], DMFF-DTA [11], and MMSG-DTA [9]. Performance was assessed using three key metrics MSE, CI, and r$_{m}^{2}$.

On the Davis dataset Table 3, KANPM-DTA achieved the best overall performance with an MSE of 0.204, CI of 0.898, and r$_{m}^{2}$ score of 0.715, surpassing all existing baselines. Specifically, KANPM-DTA reduces the MSE by 6.42% (from 0.218 to 0.204) and increases the CI by 0.45% (from 0.894 to 0.898), and the r$_{m}^{2}$ by 1.85% (from 0.702 to 0.715) compared with the second-best method, DMFF-DTA. The improvements highlight the model’s ability to capture meaningful sequence–structure interactions and effectively utilize multimodal representations.

**Table 3:** The performance of KANPM-DTA and other mainstream methods on the Davis dataset.

Model	MSE	CI	r$_{m}^{2}$
DeepDTA(2018)^a^	0.284 (0.008)	0.869 (0.002)	0.624 (0.015)
GraphDTA(2021)^a^	0.242 (0.010)	0.880 (0.001)	0.683 (0.010)
FusionDTA(2022)^a^	0.226 (0.005)	0.891 (0.001)	0.686 (0.020)
MgraphDTA(2022)^a^	0.228 (0.004)	0.883 (0.002)	0.679 (0.022)
AttentionSiteDTI(2022)^a^	0.238 (0.007)	0.886 (0.003)	0.683 (0.021)
MSGNN-DTA(2023)^a^	0.234 (0.005)	0.890 (0.002)	0.686 (0.014)
AttentionMGT-DTA(2024)^a^	0.229 (0.006)	0.888 (0.002)	0.690 (0.017)
MMFA-DTA(2024)^b^	0.219 (0.005)	0.892 (0.002)	0.688 (0.013)
DMFF-DTA(2025)^a^	0.218 (0.004)	0.894 (0.002)	0.702 (0.029)
KANPM-DTA	$\boldsymbol{0.204 (0.005)}$	$\boldsymbol{0.898 (0.001)}$	$\boldsymbol{0.715 (0.015)}$

^a,b^These results are taken from DMFF-DTA [11] and MMFA-DTA [35], respectively. The best results are highlighted in bold.

Table 4 compares the performance of KANPM-DTA with SOTA baseline methods on the KIBA dataset and achieved the best performance in each evaluation metric, reporting the lowest MSE of 0.137, the highest CI of 0.892, and an r$_{m}^{2}$ score of 0.784. Specifically, KANPM-DTA reduces the MSE by 4.86% (from 0.144 to 0.137) and increases the CI by 0.34% (from 0.889 to 0.892) and the r$_{m}^{2}$ by 0.90% (from 0.777 to 0.784) compared with the second-best method, DMFF-DTA. This demonstrates the robustness of the model across datasets with different label distributions and biological diversity.

**Table 4:** The performance of KANPM-DTA and other mainstream methods on KIBA dataset.

Model	MSE	CI	r$_{m}^{2}$
DeepDTA(2018)^a^	0.201 (0.003)	0.850 (0.002)	0.671 (0.017)
GraphDTA(2021)^a^	0.192 (0.001)	0.848 (0.002)	0.718 (0.004)
FusionDTA(2022)^a^	0.155 (0.002)	0.882 (0.001)	0.750 (0.015)
MgraphDTA(2022)^a^	0.152 (0.003)	0.884 (0.002)	0.766 (0.016)
AttentionSiteDTI(2022)^a^	0.157 (0.004)	0.881 (0.002)	0.759 (0.016)
MSGNN-DTA(2023)^a^	0.149 (0.003)	0.885 (0.002)	0.763 (0.009)
AttentionMGT-DTA(2024)^a^	0.150 (0.003)	0.879 (0.001)	0.762 (0.011)
MMFA-DTA(2024)^b^	0.148 (0.003)	0.889 (0.001)	0.777 (0.010)
DMFF-DTA(2025)^a^	0.144 (0.002)	0.889 (0.002)	0.773 (0.016)
KANPM-DTA	$\boldsymbol{0.137 (0.003)}$	$\boldsymbol{0.892 (0.001)}$	$\boldsymbol{0.784 (0.009)}$

^a,b^These results are taken from DMFF-DTA [11] and MMFA-DTA [35], respectively. The best results are highlighted in bold.

As presented in Table 5, the superior performance is consistent on the Metz dataset, with KANPM-DTA outperforming on all metrics. It decreases the MSE by 4.44% and improves the r$_{m}^{2}$ by 0.84% relative to the second-best method MMSG-DTA. Additionally, on the BindingDB dataset, KANPM-DTA outperforms the second-best method DeepDTAGen [36], with a 5.46% reduction in MSE, a 0.80% improvement in CI, and a 1.05% increase in r$_{m}^{2}$ (Table S4 in Supplementary File 1). However, KANPM-DTA achieved the best CI among all the models evaluated. The superior performance across all four datasets demonstrates the effectiveness and flexibility of the proposed framework.

**Table 5:** The performance of KANPM-DTA and other mainstream methods on Metz dataset.

Model	MSE	CI	r$_{m}^{2}$
DeepDTA(2018)^a^	0.286 (0.004)	0.815 (0.001)	0.668 (0.003)
GraphDTA(2021)^a^	0.282 (0.005)	0.815 (0.002)	0.669 (0.008)
MgraphDTA(2022)^a^	0.265 (0.002)	0.822 (0.001)	0.701 (0.001)
TDGraphDTA(2023)^a^	0.264 (0.005)	0.824 (0.005)	0.708 (0.003)
GPCNDTA(2023)^a^	0.248 (0.005)	0.834 (0.001)	0.686 (0.001)
MDCT-DTA(2024)^a^	0.278 (0.003)	0.824 (0.005)	0.701 (0.008)
LLMDTA(2025)^b^	0.329 (0.001)	0.796 (0.001)	0.642 (0.001)
MMSG-DTA(2025)^a^	0.254 (0.002)	0.826 (0.004)	0.717 (0.003)
KANPM-DTA	$\boldsymbol{0.237 (0.004)}$	$\boldsymbol{0.838 (0.002)}$	$\boldsymbol{0.723 (0.005)}$

^a,b^These results are taken from MMSG-DTA [9] and LLMDTA [32], respectively. The best results are highlighted in bold.

### Performance comparisons with baseline models under cold-start settings

To assess the generalization capacity of KANPM-DTA in real-world scenarios, where new drugs and targets are encountered during inference, we conducted experiments under three cold-start scenarios: unseen drugs, unseen proteins, and all unseen. Comparisons were made against recent strong baselines on both the Davis and KIBA datasets.

As shown in Table 6, KANPM-DTA consistently outperforms competing approaches in Davis cold start settings. In the unseen drug scenario, the model achieves relative improvements of 3.68%, 0.5%, and 16.3% in MSE, CI, and r$_{m}^{2}$, respectively, over the second-best method, PMMR. In the unseen protein scenario, KANPM-DTA further surpasses DMFF-DTA with respective gains of 4.85%, 2.02%, and 10.97% in terms of MSE, CI and r$_{m}^{2}$, respectively. In the most challenging all unseen scenario, our method demonstrates significant boosts of 11.45%, 10.68%, and 29.3% in terms of MSE, CI, and r$_{m}^{2}$ compared with other models. These results demonstrate the superior robustness and generalization ability of KANPM-DTA under more realistic generalization testing.

**Table 6:** The model performance for unseen drug, unseen protein, and all unseen on Davis dataset.

Settings	Model	MSE	CI	r$_{m}^{2}$
Unseen drug	GraphDTA(2021)^a^	0.920 (0.029)	0.678 (0.036)	0.160 (0.019)
	FusionDTA(2022)^a^	0.581 (0.094)	0.737 (0.012)	0.187 (0.034)
	MgraphDTA(2022)^a^	0.563 (0.065)	0.729 (0.022)	0.192 (0.021)
	MSGNN-DTA(2023)^a^	0.560 (0.075)	0.731 (0.036)	0.186 (0.058)
	GRA-DTA(2024)^b^	0.578 (0.005)	0.725 (0.015)	0.121 (0.024)
	PMMR(2025)^c^	0.517 (0.042)	0.794 (0.026)	0.325 (0.086)
	DMFF-DTA(2025)^a^	0.548 (0.063)	0.742 (0.037)	0.228 (0.073)
	KANPM-DTA	$\boldsymbol{0.498 (0.038)}$	$\boldsymbol{0.798 (0.021)}$	$\boldsymbol{0.378 (0.046)}$
Unseen protein	GraphDTA(2021)^a^	0.510 (0.086)	0.729 (0.012)	0.154 (0.014)
	FusionDTA(2022)^a^	0.364 (0.021)	0.826 (0.011)	0.435 (0.023)
	MgraphDTA(2022)^a^	0.359 (0.023)	0.813 (0.008)	0.425 (0.028)
	MSGNN-DTA(2023)^a^	0.361 (0.053)	0.816 (0.018)	0.430 (0.039)
	GRA-DTA(2024)^b^	0.376 (0.006)	0.827 (0.015)	0.453 (0.024)
	PMMR(2025)^c^	0.329 (0.021)	0.833 (0.013)	0.471 (0.030)
	DMFF-DTA(2025)^a^	0.330 (0.047)	0.840 (0.014)	0.501 (0.044)
	KANPM-DTA	$\boldsymbol{0.314 (0.017)}$	$\boldsymbol{0.857 (0.011)}$	$\boldsymbol{0.556 (0.023)}$
All unseen	GraphDTA(2021)^a^	0.968 (0.096)	0.579 (0.017)	0.026 (0.016)
	FusionDTA(2022)^a^	0.876 (0.091)	0.645 (0.043)	0.072 (0.048)
	MgraphDTA(2022)^a^	0.874 (0.090)	0.636 (0.021)	0.071 (0.041)
	MSGNN-DTA(2023)^a^	0.791 (0.078)	0.650 (0.053)	0.076 (0.064)
	GRA-DTA(2024)^b^	0.560 (0.007)	0.690 (0.012)	0.101 (0.024)
	PMMR(2025)^c^	0.550 (0.084)	0.683 (0.020)	0.191 (0.061)
	DMFF-DTA(2025)^a^	0.759 (0.071)	0.655 (0.050)	0.095 (0.076)
	KANPM-DTA	$\boldsymbol{0.487 (0.052)}$	$\boldsymbol{0.756 (0.016)}$	$\boldsymbol{0.247 (0.039)}$

^a,b,c^These results are taken from DMFF-DTA [11], GRA-DTA [8] and PMMR [34], respectively. The best results are highlighted in bold.

Table 7 summarizes the results on the KIBA dataset, where KANPM-DTA again demonstrates strong performance under cold-start conditions. In the unseen drug scenario, both KANPM-DTA and GRA-DTA outperform all other baselines with KANPM-DTA surpassing GRA-DTA by 4.18% for CI and 4.94% for r$_{m}^{2}$. Notably, GRA-DTA attains the lowest MSE among all evaluated methods. In the unseen protein scenario, KANPM-DTA exhibits notable improvements over DMFF-DTA, with gains of 25.85%, 4.41%, and 19.06% in terms of MSE, CI, and r$_{m}^{2}$, respectively. In the most challenging all unseen scenario, our method demonstrates significant boosts of 3.45% and 32.63% in terms of CI, and r$_{m}^{2}$ compared with other models. On the other hand, GRA-DTA achieved the best MSE performance among all the models evaluated.

**Table 7:** The model performance for unseen drug, unseen protein, and all unseen on KIBA dataset.

Settings	Model	MSE	CI	r$_{m}^{2}$
Unseen drug	GraphDTA(2021)^a^	0.471 (0.047)	0.713 (0.002)	0.342 (0.007)
	FusionDTA(2022)^a^	0.429 (0.031)	0.748 (0.005)	0.364 (0.012)
	MgraphDTA(2022)^a^	0.425 (0.047)	0.746 (0.002)	0.366 (0.016)
	MSGNN-DTA(2023)^a^	0.426 (0.043)	0.747 (0.003)	0.358 (0.022)
	GRA-DTA(2024)^b^	$\boldsymbol{0.337 (0.007)}$	0.765 (0.012)	0.466 (0.024)
	DMFF-DTA(2025)^a^	0.408 (0.036)	0.753 (0.006)	0.397 (0.020)
	KANPM-DTA	0.351 (0.014)	$\boldsymbol{0.797 (0.004)}$	$\boldsymbol{0.489 (0.022)}$
Unseen protein	GraphDTA(2021)^a^	0.469 (0.089)	0.610 (0.035)	0.368 (0.057)
	FusionDTA(2022)^a^	0.439 (0.062)	0.685 (0.032)	0.390 (0.067)
	MgraphDTA(2022)^a^	0.435 (0.055)	0.674 (0.028)	0.382 (0.047)
	MSGNN-DTA(2023)^a^	0.438 (0.061)	0.683 (0.025)	0.399 (0.054)
	GRA-DTA(2024)^b^	0.435 (0.007)	0.692 (0.012)	0.355 (0.024)
	DMFF-DTA(2025)^a^	0.410 (0.063)	0.748 (0.053)	0.446 (0.064)
	KANPM-DTA	$\boldsymbol{0.304 (0.035)}$	$\boldsymbol{0.781 (0.019)}$	$\boldsymbol{0.531 (0.041)}$
All unseen	GraphDTA(2021)^a^	0.676 (0.113)	0.601 (0.030)	0.149 (0.067)
	FusionDTA(2022)^a^	0.587 (0.086)	0.641 (0.023)	0.193 (0.053)
	MgraphDTA(2022)^a^	0.590 (0.094)	0.626 (0.028)	0.182 (0.012)
	MSGNN-DTA(2023)^a^	0.581 (0.079)	0.648 (0.038)	0.180 (0.021)
	GRA-DTA(2024)^b^	$\boldsymbol{0.441 (0.007)}$	0.657 (0.012)	0.228 (0.024)
	DMFF-DTA(2025)^a^	0.567 (0.082)	0.667 (0.035)	0.236 (0.037)
	KANPM-DTA	0.542 (0.041)	$\boldsymbol{0.690 (0.014)}$	$\boldsymbol{0.313 (0.011)}$

^a,b^These results are taken from DMFF-DTA [11] and GRA-DTA [8], respectively. The best results are highlighted in bold.

**Table 8:** Ablation evaluation results for different components of KANPM-DTA on the Davis dataset.

Settings	Experiments	MSE	CI	r$_{m}^{2}$
Warm	W/O Graph	0.227 (0.008)	0.893 (0.002)	0.676 (0.016)
	W/O Sequence	0.540 (0.015)	0.794 (0.004)	0.331 (0.028)
	W/O Linear Attention	0.250 (0.006)	0.886 (0.003)	0.629 (0.014)
	W/O Gated Fusion	0.229 (0.008)	0.891 (0.002)	0.655 (0.018)
	W/O ESM-C Node Features	0.224 (0.009)	0.884 (0.006)	0.696 (0.024)
	W/O KAN	0.221 (0.008)	0.889 (0.006)	0.701 (0.021)
	Ours	$\boldsymbol{0.204 (0.005)}$	$\boldsymbol{0.898 (0.001)}$	$\boldsymbol{0.715 (0.015)}$
Unseen drug	W/O Graph	0.741 (0.032)	0.716 (0.018)	0.169 (0.012)
	W/O Sequence	0.704 (0.028)	0.759 (0.015)	0.145 (0.010)
	W/O Linear Attention	0.584 (0.025)	0.789 (0.016)	0.281 (0.020)
	W/O Gated Fusion	0.522 (0.035)	0.763 (0.019)	0.331 (0.031)
	W/O ESM-C Node Features	0.589 (0.027)	0.745 (0.017)	0.284 (0.021)
	W/O KAN	0.509 (0.030)	0.788 (0.018)	0.357 (0.026)
	Ours	$\boldsymbol{0.498 (0.038)}$	$\boldsymbol{0.798 (0.021)}$	$\boldsymbol{0.378 (0.046)}$
Unseen protein	W/O Graph	0.328 (0.014)	0.838 (0.090)	0.521 (0.022)
	W/O Sequence	0.528 (0.022)	0.793 (0.011)	0.310 (0.019)
	W/O Linear Attention	0.368 (0.016)	0.827 (0.010)	0.483 (0.021)
	W/O Gated Fusion	0.360 (0.015)	0.823 (0.010)	0.507 (0.022)
	W/O ESM-C Node Features	0.328 (0.014)	0.851 (0.008)	0.521 (0.023)
	W/O KAN	0.355 (0.015)	0.845 (0.009)	0.533 (0.023)
	Ours	$\boldsymbol{0.314 (0.017)}$	$\boldsymbol{0.857 (0.011)}$	$\boldsymbol{0.556 (0.023)}$
All unseen	W/O Graph	0.638 (0.041)	0.668 (0.025)	0.101 (0.008)
	W/O Sequence	0.612 (0.038)	0.721 (0.022)	0.096 (0.007)
	W/O Linear Attention	0.586 (0.036)	0.619 (0.028)	0.098 (0.007)
	W/O Gated Fusion	0.526 (0.034)	0.679 (0.024)	0.197 (0.015)
	W/O ESM-C Node Features	0.794 (0.045)	0.659 (0.027)	0.097 (0.007)
	W/O KAN	0.742 (0.043)	0.667 (0.026)	0.112 (0.009)
	Ours	$\boldsymbol{0.487 (0.052)}$	$\boldsymbol{0.756 (0.016)}$	$\boldsymbol{0.247 (0.039)}$

The best results are highlighted in bold.

Additionally, we examined the existence of cold-start scenario by evaluating the similarity between drugs and proteins across both the training and test sets. This evaluation is crucial for assessing the model’s generalization capabilities. The average of the maximum similarity values between each test-train drug or protein pair across different folds is summarized in Tables S6–S8 in Supplementary File 1. As shown in the tables, in some cases, the average maximum similarity values for the test-train drug or protein pairs exceed the standard cutoff thresholds. In practice, maximum similarity values only occur for a small subset of pairs, while the majority of similarity values remain within the standard cutoff ranges. Another consideration is that both the Davis and KIBA datasets are strongly kinase-centric, which naturally leads to relatively high similarity between the test and training sets. This can be considered as a limitation associated with the kinase-focused data and experimental design. In this work, the test sets are treated as unseen in the sense that they do not share identical drugs or proteins with the training sets, although they do not fully satisfy the stricter definition of cold-start that requires lower similarity thresholds. All findings confirm that the proposed multimodal integration and novel protein graph construction approach significantly enhance model generalization in cold-start scenarios, making KANPM-DTA a reliable framework for practical drug discovery applications where novel molecules and targets are frequently encountered.

### Ablation study

#### The effect of model architecture modules

To evaluate the effect of each components in our proposed KANPM-DTA framework, we construct six variants based on our model for ablation experiments on Davis datasets.

- W/O Graph: Using only protein sequence and drug strings as inputs and pretrained models were used to extract features.
- W/O Sequence: Using only sequence to construct molecular graph and protein graph and GNNs were employed to extract graph-based features.
- W/O Linear Attention: Removing the linear attention mechanism from the model architecture and using concatenation of drug and protein features.
- W/O Gated Fusion: Removing the gated fusion module and using concatenation to combine different feature representations.
- W/O ESM-C Node Features: Not using ESM-C embeddings as node features in the graph construction.
- W/O KAN: Replacing the KAN with standard MLP layers in the model.

Table 8 presents the comparison results, and the waterfall chart of MSE gains when a particular component is removed from the KANPM-DTA framework is shown in Figure S2 in Supplementary File 1. Removing graph features leads to the worst performance in both warm and cold-start scenarios, with MSE increases of 11.27%, 48.99%, 4.45%, and 30.99%, respectively. This highlights the critical importance of graph-based features for accurately modeling DTIs. In the W/O Sequence, a notable decline in performance is observed across all four scenarios, underscoring the enhanced generalization ability of the model when combining 1D sequence-based pretrained features with GNN-driven features.

For the W/O Linear Attention, we observe a notable increase in MSE and decrease in CI and r$_{m}^{2}$ across all four settings. This indicates that the inclusion of linear attention enhances the model’s generalization capability for unseen drugs and proteins, outperforming the simple concatenation approach. Additionally, the W/O Gated Fusion configuration shows reduced performance across all four settings, indicating that concatenating independent graph features for drugs and targets is less effective than using the fused representation generated by the gated fusion module. Excluding ESM-C node features leads to a significant decline in performance compared with the full model, emphasizing the crucial role of ESM-C node feature extraction in enhancing the generalizability of DTA predictions. These results highlight that ESM-C provides informative residue-level representations that enrich the model’s ability to capture structural and functional details within the protein graph, thus improving binding affinity predictions. Furthermore, excluding the KAN module from the full model results in a substantial performance decline across all settings, particularly in all unseen scenarios. This emphasizes KAN’s pivotal ability to approximate complex nonlinear functions more effectively than standard MLPs, a capability, i.e. critical to improving the accuracy of final affinity predictions. Moreover, training and validation learning curves for the KAN and MLP modules in the KANPM-DTA framework on the KIBA dataset are visualized in Figure S3 in Supplementary File 1, which illustrates, with KAN, the model learns more stably and generalizes better over time compared with MLP, achieving superior performance across all three metrics (MSE, CI, and $r_{m}^{2}$).

Overall, the ablation results validate the importance of each component in enhancing the accuracy and generalization capacity of the KANPM-DTA model.

#### The effect of protein language models and drug language models

**Figure 3:**
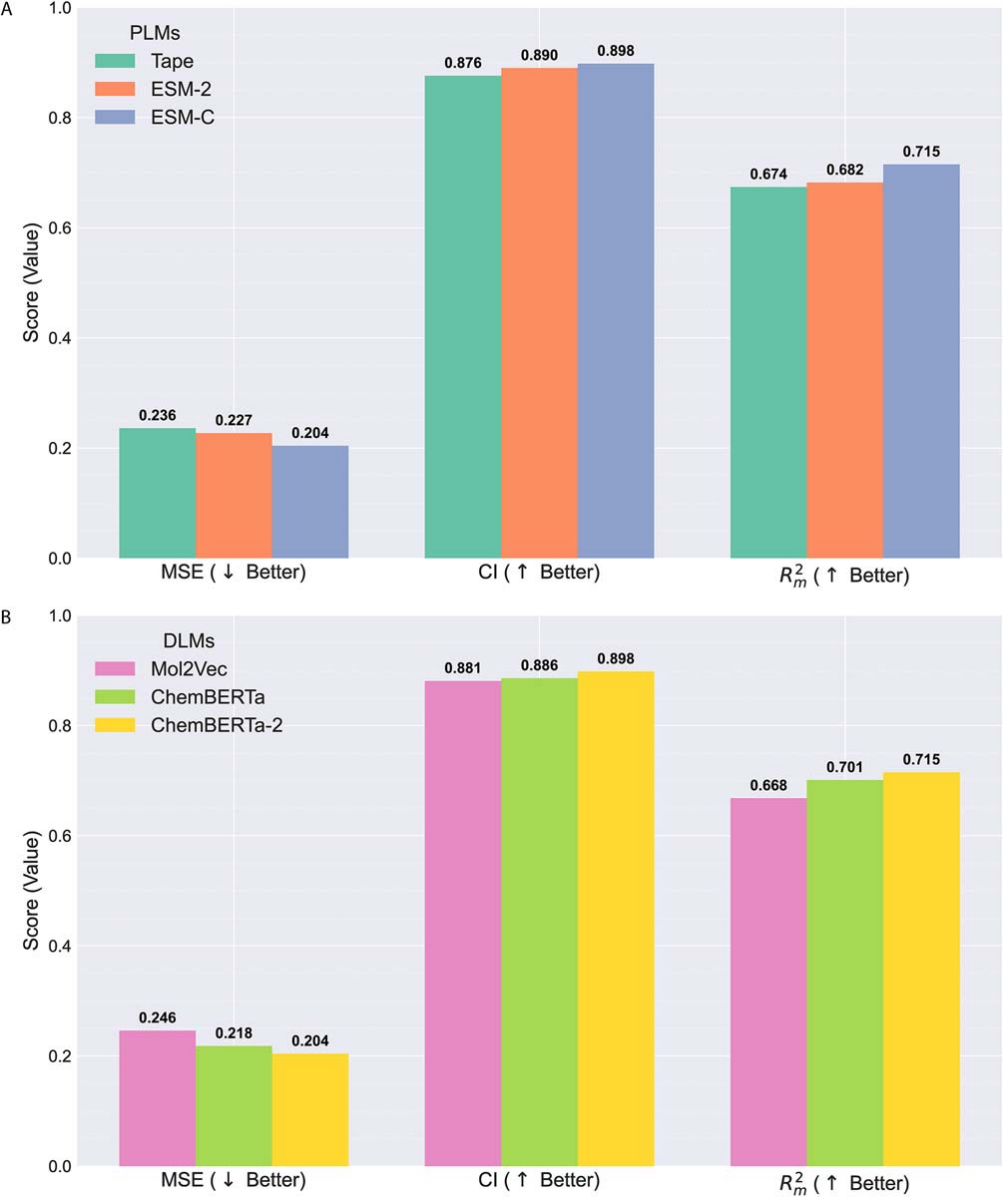
Ablation comparison of KANPM-DTA on the Davis dataset under different pretraining schemes: (A) PLMs and (B) DLMs.

In this study, we investigated the effect of different protein language models (PLMs) and drug language models (DLMs) within the KANPM-DTA framework to evaluate how pretrained representations affect the prediction of drug–target binding affinity. Specifically, we compared three PLMs, including Tape, ESM-2, and the contact-aware model ESM-C, as well as three DLMs, including Mol2Vec, ChemBERTa, and ChemBERTa-2 on the Davis dataset in terms of MSE, CI, and $r_{m}^{2}$. As shown in Figure 3A and B, using ESM-C and ChemBERTa-2 as a pretraining methods for protein and drug achieved the best performance in all metrics. In summary, the residue-level embedding of ESM-C captures evolutionary and structural information, while the labeled embedding of ChemBERTa-2 preserves the global and local chemical contexts. This combination outperforms other PLMs and DLMs, demonstrating superior generalization for unseen drug–target pairs.

## Case study

To further evaluate the generalization capacity of KANPM-DTA in structure-free virtual screening, we investigated its ability to identify DTIs involving the EGFR. EGFR is a membrane-bound tyrosine kinase, i.e. frequently over-expressed in multiple cancers. Therapeutic inhibition of EGFR is a well-established strategy in the treatment of EGFR-driven malignancies, including non-small-cell lung cancer and pancreatic cancer. We screened 3137 FDA-approved small molecules from the DrugBank database, none of which were included in the KIBA training dataset. Binding affinity was predicted in terms of KIBA scores, and the top 12 ranked compounds are summarized in Table 9. Notably, four of the highest-scoring compounds dacomitinib, lapatinib, gefitinib, and afatinib are clinically validated EGFR inhibitors, supporting the model’s predictive validity. The structurally analogous compounds corresponding to the top-ranked 12 compounds in the KIBA dataset were examined using a pairwise Tanimoto similarity coefficient based on Morgan fingerprints. No significant similarity was found between them (Avg. Tc < 0.15), demonstrating that the model possesses true generalization capabilities for unseen drug–target pairs. The detailed results of the Tanimoto similarity calculation are provided in Supplementary File 2.

**Table 9:** Top-12 predicted potential drugs for EGFR based on predicted KIBA score.

Rank	Drugs	DrugBank ID	KIBA score	Evidence
1	Dacomitinib	DB11963	13.55	DrugBank
2	Lapatinib	DB01259	13.52	DrugBank
3	Gefitinib	DB00317	13.47	DrugBank
4	Afatinib	DB08916	13.40	DrugBank
5	Staurosporine	DB02010	13.36	[37]
6	Emodin	DB07715	13.29	–
7	Tenofovir alafenamide	DB09299	13.23	–
8	Kaempferol	DB01852	13.11	[38–40]
9	Genistein	DB01645	13.05	[41,42]
10	Apigenin	DB07352	12.92	–
11	Idarubicin	DB01177	12.90	–
12	Astemizole	DB00637	12.80	–

**Figure 4:**
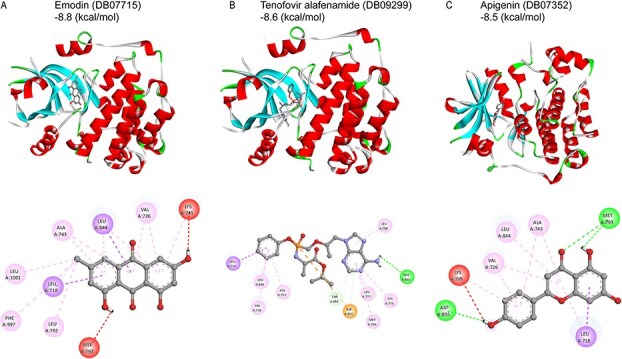
Binding interactions between epidermal growth factor receptor (EGFR) (Protein Data Bank (PDB) ID: 3POZ) and drug molecules predicted by KANPM-DTA, highlighting key interacting residues.

Although the model predicts some drugs have no direct association with EGFR-targeted therapies, further validation of EGFR–drug interactions was carried out through molecular docking experiments using the crystal structure of EGFR with PDB ID 3POZ (UniProt ID: P00533) from the PDB. Docking was performed via AutoDock, and compounds demonstrating binding free energies $< -8.3$ kcal/mol, indicating high binding affinity with EGFR. Staurosporine, emodin, tenofovir alafenamide, kaempferol, genistein, apigenin, idarubicin, and astemizole met this threshold, with idarubicin, staurosporine, and astemizole showing especially strong predicted interactions. Molecular interactions were further visualized using PyMOL [43], highlighting key hydrogen bond formations between each ligand and the EGFR binding site Figure 4 and Figure S4 in Supplementary File 1. To ensure the reliability of our predicted binding affinities, we have incorporated known active and inactive compounds targeting EGFR as internal references to calibrate and contextualize the docking scores. The inclusion of these reference compounds ensures that our docking scores are appropriately contextualized and allow for meaningful comparisons. The results for these compounds are summarized in Table S5 in Supplementary File 1. As shown, the docking scores for the active compounds are consistent with known binding ranges, while the docking scores for the inactive compounds are in line with expected low binding affinities. Furthermore, the predicted affinities for the novel compounds align closely with the expected binding ranges based on the known active compounds, demonstrating the reliability and robustness of our model. These results demonstrate that KANPM-DTA can reliably generalize to unseen compounds and may serve as an effective tool for structure-free virtual screening in drug repurposing pipelines.

## Conclusion

In this work, we proposed KANPM-DTA, a graph–sequence integrated framework with KAN for robust and generalizable drug–target affinity prediction. Unlike traditional end-to-end DTA models, KANPM-DTA leverages pretrained ChemBERTa-2 and ESM-C for drug and protein sequence representations, while incorporating ESM-2–guided protein graph construction. These pretrained features, combined with gated fusion and linear attention mechanisms, enable the model to capture both structural and sequential dependencies, thereby enhancing its generalization capability. Experimental results on four widely used benchmark datasets demonstrate that KANPM-DTA consistently outperforms existing state-of-the-art methods in both warm- and cold-start settings. Particularly under novel drug and novel protein scenarios, the model achieves superior prediction performance, highlighting its robustness for real-world applications. Furthermore, the case study on EGFR illustrates the framework’s potential in identifying candidate drugs and supporting drug repurposing efforts, with several predicted compounds aligning with known therapeutic evidence.

Our study represents a successful practice of integrating pretrained biological language models, graph encoders, and KAN regression for computational drug discovery. With the rapid advancement of pretrained models and multimodal integration strategies, we believe the cold-start challenge in DTA prediction will be increasingly mitigated. Future work will explore incorporating additional biological data sources such as pathways and structural annotations, adopting domain-specific pretraining, as well as explainability techniques to provide deeper insights into DTI mechanisms.

**Key Points**

- We introduce an ESM-guided graph formulation for drug–target affinity prediction, using ESM-2 contact maps for protein edges and ESM-C embeddings for node features to enhance understanding of protein interactions.
- We integrate protein and drug graph features through a gated fusion module, effectively capturing both intra- and inter-sequence dependencies through linear attention mechanisms.
- We introduce Kolmogorov–Arnold network for final affinity prediction, enhancing the model’s expressiveness, interpretability, and outperforming traditional multi-layer perceptrons.
- We perform comprehensive experiments on public datasets to assess the effectiveness of the proposed model.

## Supplementary Material

KANPM_DTA_Supplementary_File_1_bbag112

## Data Availability

The source code and data of this study are available at the following GitHub repository: https://github.com/khanonuvov/KANPM-DTA.
