# Supplementary material for: KANPM-DTA: improving drug–target affinity prediction with Kolmogorov–Arnold networks and pretrained models
Source: Brief Bioinform. 2026 Mar 12;27(2):bbag112. doi: 10.1093/bib/bbag112 (PMC12981676; doi:10.1093/bib/bbag112)
Supplement: KANPM_DTA_Supplementary_File_1_bbag112 [file kanpm_dta_supplementary_file_1_bbag112.pdf]

Supplementary Materials for ‘**KANPM-DTA: Improving Drug-Target Affinity Prediction with Kolmogorov-Arnold Networks and Pre-trained Models**’

MD Youshuf Khan Rakib[1], Muhammad Habibulla Alamin[1], Jiamu Li[2], Sheikh Sohan Mamun[1], Kaleb Amsalu Gobena[1], and Shengbing Ren[1*]

[1]School of Computer Science and Engineering, Central South University, Changsha 410083, Hunan, China
[2]School of Computer Science and Engineering, Macau University of Science and Technology, Taipa 999078, Macau, China
[*]Corresponding Author: rsb@csu.edu.cn

Abstract

Accurate drug–target affinity (DTA) prediction is critical for drug discovery and repurposing. However, existing models often struggle with generalizing to unseen drug-target pairs, lack interpretability, and fail to integrate heterogeneous biological features effectively. To overcome these challenges, we introduce KANPM-DTA, a deep learning framework designed to capture richer biochemical interactions and improve prediction reliability. Specifically, an ESM-guided protein graph construction strategy incorporates evolutionary and structural information to overcome underexplored protein representations. A gated fusion mechanism was employed to integrate drug–protein graph features, while linear attention captures cross-modal dependencies that enhance discriminative power. For the final affinity prediction, a Kolmogorov-Arnold Network (KAN) was used, offering a stronger nonlinear approximation and improved interpretability. Comprehensive experiments on benchmark datasets demonstrate that KANPM-DTA significantly outperforms state-of-the-art (SOTA) methods. On the Davis, KIBA, Metz and BindingDB datasets, we achieved significant performance improvements under warm setting, with MSE reductions of 6.42%, 4.86%, 4.44%, and 5.46%, CI increases of 0.45%, 0.34%, 0.48% and 0.80%, and r_m^2 gains of 1.85%, 0.90%, 0.84%, and 1.05%, respectively. Moreover, a case study on the epidermal growth factor receptor (EGFR) further highlights the effectiveness of KANPM-DTA in predicting DTAs for unknown drug-target pairs, emphasizing its potential for real-world applications in drug discovery. However, wet-lab validation is required to assess the applicability of the results.

Keywords: Drug Target Affinity, Graph Neural Networks, Attention Mechanisms, Pre-trained Models, Kolmogorov-Arnold Networks.

This pdf file includes:

- Supplementary Methods and Materials
- Supplementary Results
- Supplementary Tables S1–S8
- Supplementary Figures S1–S4

1 Pretrained Model Variants

In this work, we employ three types of pretrained models to extract prior knowledge from molecular and protein sequences: (I) a ChemBERTa-2 for drug sequence embeddings, (II) an ESM-2 model for protein contact-map prediction, and (III) an ESM-C model for protein sequence embeddings.

In our framework, we rely on three families of pretrained models for drug and protein representations:

- ChemBERTa-2. We used the RoBERTa-based ChemBERTa variant released as `DeepChem/ChemBERTa-77M-MTR` to obtain SMILES-level drug embeddings.
- ESM-2. For residue–residue contact probabilities, we use the ESM-2 model `esm2_t36_3B_UR50D` with its built-in contact prediction head.
- ESM-C. For protein residue embeddings, we use the ESM-C model `esmc_600m` as the backbone encoder.

2 Details of KAN Block

2.1 KAN Architecture and Specification

The KAN block is implemented as a three-layer network that processes the 512-dimensional feature vector Z before mapping to the final scalar output $\hat{y}$.

The core computational unit of the KAN is the KANLinear layer, where the transformation between input x and output y is defined by a weighted sum of parametrized one-dimensional functions $f_{i,j}(x)$:

$$y_j = \sum_{i=1}^{N_{\text{in}}} f_{i,j}(x_i) \tag{1}$$

Each function $f_{i,j}(x)$ is parameterized by the sum of a base activation term $\sigma_{\text{base}}(x)$ and a B-spline component $B_k(x)$:

$$f_{i,j}(x) = w_{\text{base}} \cdot \sigma_{\text{base}}(x) + \sum_{k=1}^{G+K} c_k \cdot B_k(x) \tag{2}$$

The base activation $\sigma_{\text{base}}(x)$ is the SiLU. A schematic of the three-layer KAN block architecture is provided in Supplementary Fig. S1, and the full set of hyperparameters, including layer dimensions, spline order, grid size is detailed in Supplementary Table S2.

2.2 KAN Initialization and Regularization

The base weights and spline scalers are initialized using the Kaiming Uniform distribution. The spline coefficients are initialized by fitting the B-spline basis functions to a curve derived from small random noise, which ensures the starting function is smooth and promotes stable training.

To enforce feature sparsity and enhance generalization capacity, a composite regularization loss $\mathcal{L}_{\text{reg}}$ is applied to the spline coefficients w_{spline}:

$$\mathcal{L}_{\text{reg}} = \lambda_1 \sum_{\ell=1}^{L} \|w_{\text{spline}}^{(\ell)}\|_1 + \lambda_2 \sum_{\ell=1}^{L} \sum_{i,j} (P_{i,j} \log P_{i,j}) \tag{3}$$

This loss combines two terms: I) $\mathbf{L}_1$ regularization λ_1 on the spline coefficients to encourage sparsity, and II) Entropy regularization λ_2 based on the normalized L_1 magnitude $P_{i,j}$, which promotes a balanced use of features.

3 File Preparation for Molecular Docking

Molecular docking studies are performed for the unconfirmed drug-target interactions predicted by KANPM-DTA. The high-resolution crystallographic structure of the target protein, such as EGFR (PDB ID: 3POZ), is obtained from the RCSB PDB database. The docking process is carried out using AutoDock Vina. The following steps outline the process:

- Download the 3D structures of the compounds in SDF format from PubChem based on their SMILES strings. Convert the structures into .pdb format using PyMol for further processing with AutoDockTools.
- Save the compound structures in .pdbqt format using AutoDockTools.
- Download the 3D structure of the target protein in .pdb format from PDB. Using AutoDockTools, remove water molecules, add hydrogen atoms, assign Kollman charges, and save the processed protein structure in format of .pdbqt.
- The active sites of the target protein are predicted using P2Rank, a tool designed for reliable active site prediction based on protein structure. Once the binding pocket (active site) is identified, docking grids are generated centered on this active site to focus the docking process on the relevant region of the protein.
- Create a configuration file that includes the grid specifications and initiates the molecular docking for the top unconfirmed drug candidates. Rotatable bonds are allowed for the compounds during the docking process. The configuration details of the docking grids are provided in Supplementary Table S3.

Table S1. Node features for a drug graph [5].

Feature Name	Dimension
Atom Symbols	44
Formal Charge	1
Explicit Valence	1
Number of Atomic	1
Hybridization Type	3
Whether the atom is Donor	1
Degree of the atom (one-hot)	11
Number of Radical Electrons	1
Whether the atom is Acceptor	1
Whether the atom is Aromatic	1
Number of Explicit Hydrogens	1
Total number of H atoms bound to the heavy atom	11
Number of implicit H atoms bound to the heavy atom	11
Total	88

Table S2. Detailed Hyperparameter Settings of the KAN Prediction Head.

Hyperparameter	Value
Layer Dimensions (Hidden)	$[512, 1024, 512, 1]$
Spline Order (K)	3
Grid Size (G)	5
Base Activation (σ_{base})	SiLU
Initialization Scale (Base)	1.0
Initialization Scale (Spline)	1.0
Initialization Scale (Noise)	0.1
Grid Initial Range	$[-1, 1]$
Adaptive Grid Weight (ϵ_{grid})	0.02
L_1 Regularization Weight (λ_1)	1.0*
Entropy Regularization Weight (λ_2)	1.0*

* These values are the default settings in the KAN implementation.

Table S3. The configurations of the grids and docking parameters.

Parameter	Value
Center X	16.660
Center Y	32.301
Center Z	9.294
Size X	68
Size Y	54
Size Z	52
Exhaustiveness	10
Receptor	EGFR.pdbqt
PDB ID	3POZ

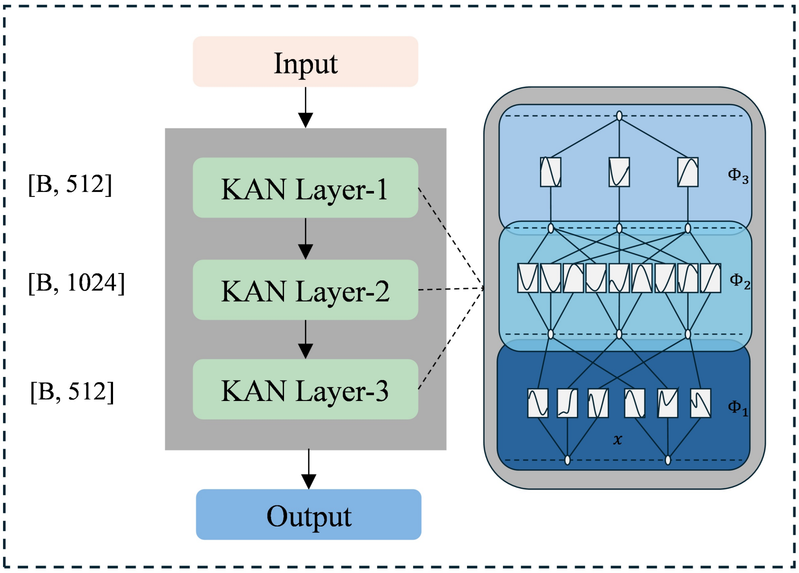

Fig. S1. The KAN block architecture.

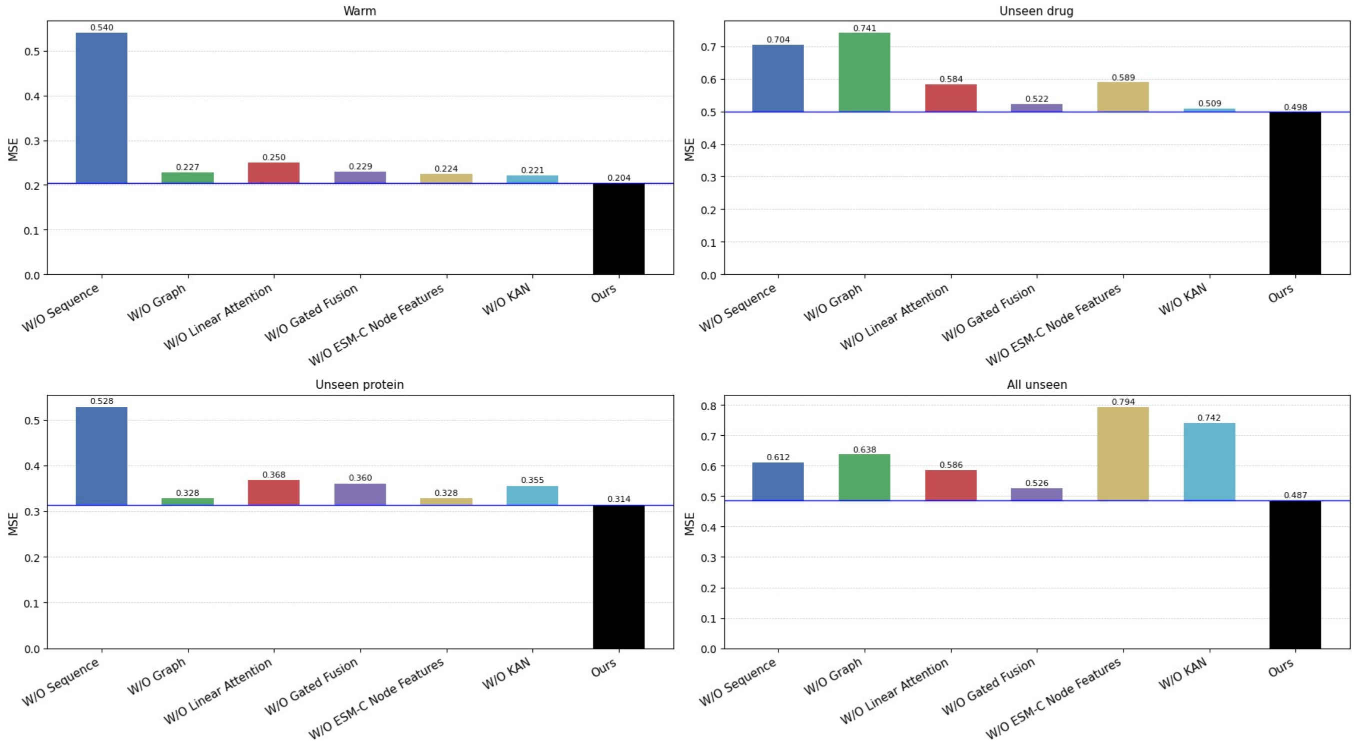

Fig. S2. Waterfall charts of MSE gains for KANPM-DTA on the Davis dataset under the warm, unseen drug, unseen protein, and all unseen settings. Each colored bar corresponds to an ablated variant ("W/O Sequence", "W/O Graph", "W/O Linear Attention", "W/O Gated Fusion", "W/O ESM-C Node Features", "W/O KAN"), and the black bar denotes the full model ("Ours"); the vertical gap above the "Ours" baseline indicates the increase in MSE when the corresponding component is removed, highlighting its contribution to overall performance.

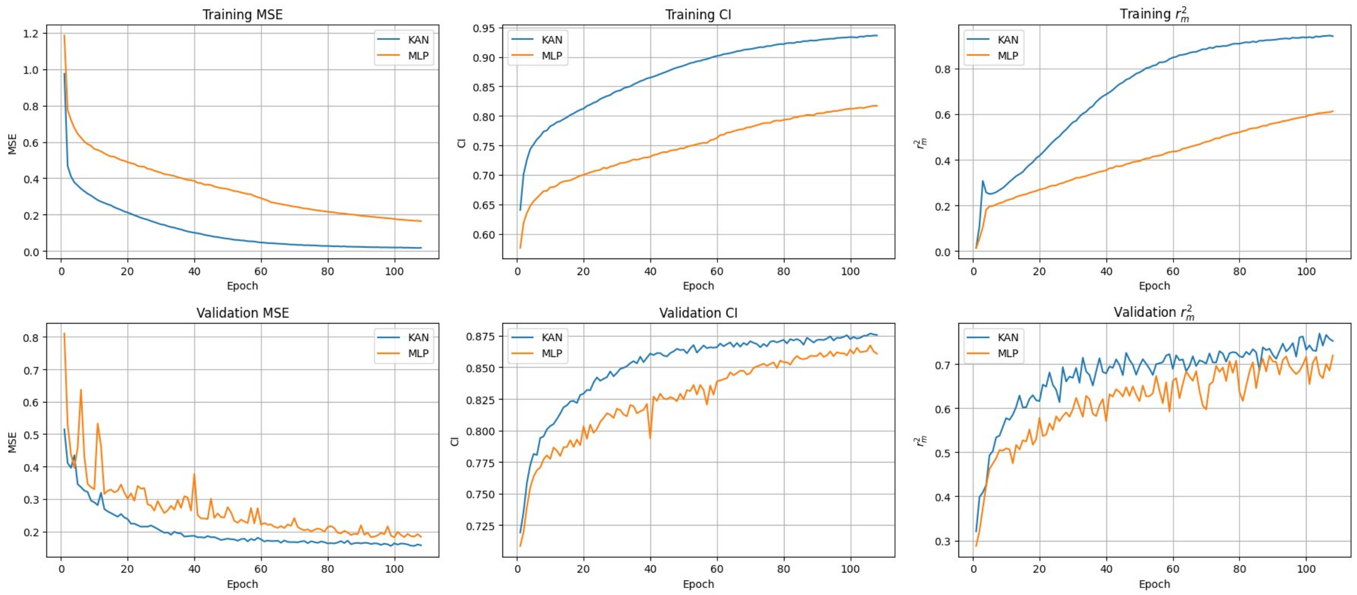

Fig. S3. Training and validation learning curves for KAN and MLP models across all three performance metrics (MSE, CI, and r_m^2) on KIBA dataset. The top row illustrates the training performance, while the bottom row shows the validation performance.

Table S4. The performance of KANPM-DTA and other mainstream methods on the BindingDB dataset. The best results are highlighted in bold.

Model	MSE	CI	r_m^2
DeepDTA(2018)[a]	0.633 (0.0002)	0.844 (0.003)	0.633 (0.004)
AttentionDTA(2022)[a]	0.745 (0.0008)	0.542 (0.001)	-
GraphDTA(GIN)(2021)[a]	0.535 (0.008)	0.858 (0.004)	-
CoVAE(2021)[a]	0.512 (0.009)	0.847 (0.00012)	0.412 (0.0008)
DeepCDA(2020)[a]	0.848 (-)	0.722 (0.01)	0.531 (0.02)
ELECTRA-DTA(2022)[a]	0.650 (-)	0.837 (0.004)	0.670 (0.012)
DoubleSG-DTA(2023)[a]	0.533 (-)	0.862 (0.002)	0.726 (0.009)
GDilatedDTA(2024)[a]	0.483 (0.002)	0.868 (0.001)	0.730 (0.001)
MF-DTA(2025)[b]	0.569 (-)	0.865 (0.001)	0.737 (0.001)
DeepDTAGen(2025)[a]	0.458 (0.002)	0.876 (0.004)	0.760 (0.003)
KANPM-DTA	**0.433(0.002)**	**0.883(0.001)**	**0.768(0.012)**

[a,b]These results are taken from DeepDTAGen [7] and MF-DTA [8], respectively.

Table S5. Validation of docking protocol for novel compounds compared with active/inactive compounds targeting EGFR.

Category	Compound	Docking Score (kcal/mol)	Reference
Active	Dacomitinib	-9.8	CHEMBL2105719
	Lapatinib	-10.8	CHEMBL554
	Gefitinib	-8.6	CHEMBL939
	Afatinib	-9.1	CHEMBL1173655
Novel	Staurosporine	-9.8	-
	Emodin	-8.8	-
	Tenofovir alafenamide	-8.6	-
	Kaempferol	-8.3	-
	Genistein	-8.4	-
	Apigenin	-8.5	-
	Idarubicin	-10.2	-
	Astemizole	-9.6	-
Inactive	Cyclophosphamide	-4.4	CHEMBL1200796
	Ciclopirox	-6.3	CHEMBL1413
	Gemfibrozil	-6.4	CHEMBL457

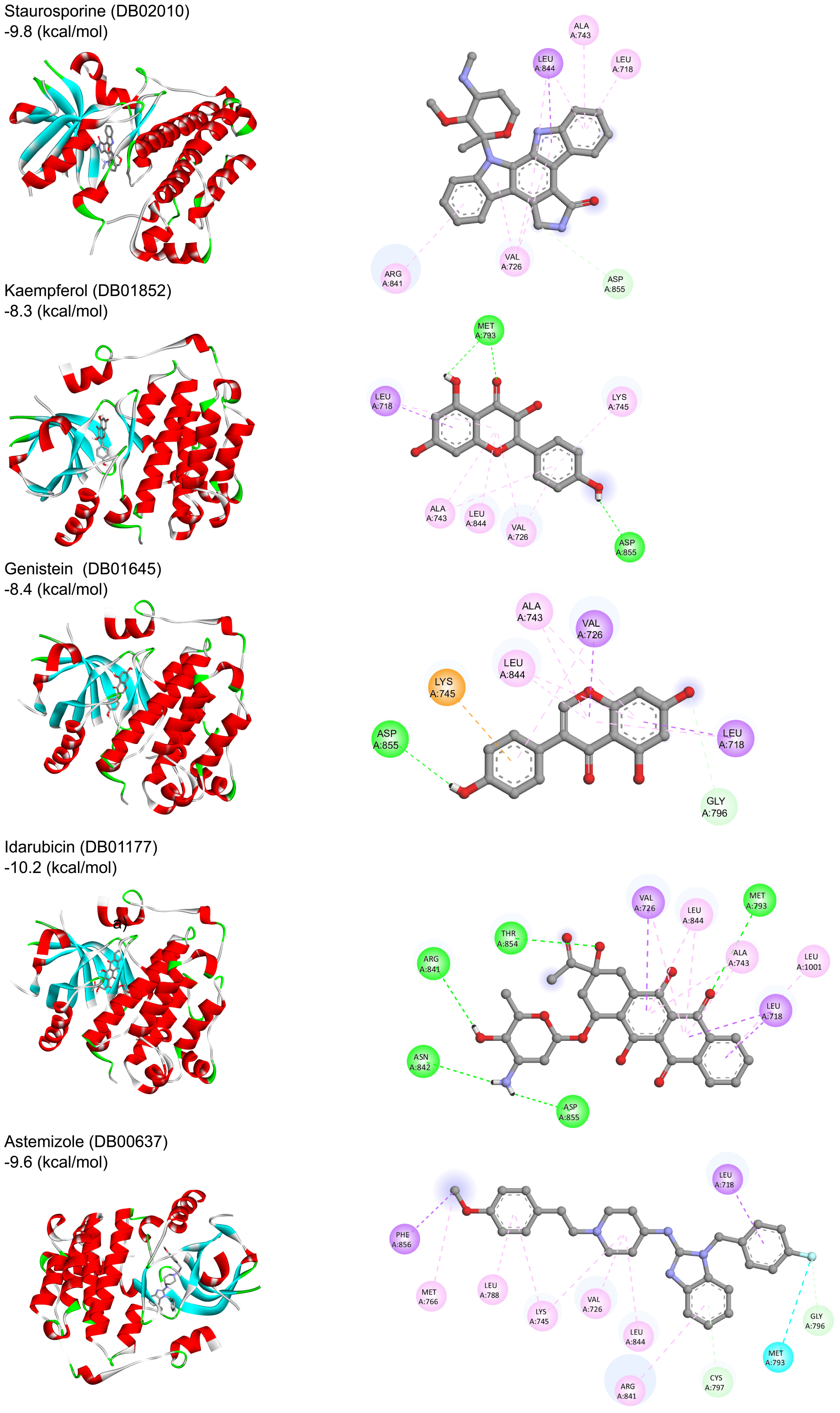

Fig. S4. Binding interactions between EGFR (PDB ID: 3POZ) and drug molecules predicted by KANPM-DTA, highlighting key interacting residues.

4 Similarity Calculation between Test and Training Sets

To calculate the similarity between the test and training sets for both drugs and proteins, we perform the following steps:

Step 1

- **Pairwise Similarity Calculation for drugs:** We perform the Tanimoto Similarity calculation for each drug in the test set by comparing it with every drug in the training set. The Tanimoto Similarity is computed by comparing the molecular fingerprints of the test and training drugs, which measure their structural similarity.
- **Pairwise Similarity Calculation for proteins**: We compute the global sequence identity for each protein in the test set by comparing it with every protein in the training set using a global pairwise alignment implemented with Biopython PairwiseAligner in global mode. After obtaining the optimal global alignment between two protein sequences, we calculate sequence identity as the fraction of exactly matched residues over the alignment length, which provides a similarity score between protein sequences.

Step 2

- **Identify Maximum Similarity:** For each drug or protein in the test set, we identify the highest similarity score when compared to all the drugs or proteins in the training set. This allows us to find the drug or protein in the training set that is most similar to each test drug or protein.

Step 3

- **Repeat for All Test Drugs or Proteins:** We repeat the previous steps for all drugs or proteins in the test set, obtaining a maximum similarity value for each test drug or protein when compared to the training drugs or proteins.
- **Average the Maximum Similarities:** After computing the maximum similarity for each drug or protein, we calculate the average of these maximum values. This provides an overall measure of how similar, on average, the drugs or proteins in the test set are to the drugs or proteins in the training set.

By performing these steps, we obtain a comprehensive measure of similarity for both drugs and proteins in Table S6-S8, which allows us to make a complete unseen test set for cold-start experiments.

Table S6. Summary statistics of drug similarity for each fold between the training and test sets under the unseen drug cold-start scenario.

Dataset	Fold 1			Fold 2			Fold 3			Fold 4			Fold 5		
	Max	Min	Mean	Max	Min	Mean	Max	Min	Mean	Max	Min	Mean	Max	Min	Mean
Davis	0.746	0.187	0.406	0.428	0.220	0.293	0.746	0.197	0.340	0.597	0.257	0.426	0.516	0.180	0.299
KIBA	0.774	0.216	0.621	0.779	0.200	0.644	0.794	0.240	0.660	0.809	0.208	0.626	0.812	0.229	0.638

Table S7. Summary statistics of protein similarity for each fold between the training and test sets under the unseen protein cold-start scenario.

Dataset	Fold 1			Fold 2			Fold 3			Fold 4			Fold 5		
	Max	Min	Mean	Max	Min	Mean	Max	Min	Mean	Max	Min	Mean	Max	Min	Mean
Davis	0.745	0.241	0.460	0.834	0.231	0.503	0.867	0.231	0.554	0.867	0.231	0.494	0.875	0.252	0.544
KIBA	0.837	0.238	0.578	0.835	0.233	0.541	0.825	0.234	0.476	0.834	0.234	0.560	0.828	0.239	0.480

Table S8. Summary statistics of drug and protein similarity for each fold between the training and test sets under the all-unseen cold-start scenario.

Dataset	Fold 1			Fold 2			Fold 3			Fold 4			Fold 5		
	Max	Min	Mean	Max	Min	Mean	Max	Min	Mean	Max	Min	Mean	Max	Min	Mean
Davis (D)	0.746	0.187	0.415	0.428	0.215	0.299	0.746	0.197	0.371	0.697	0.257	0.457	0.746	0.185	0.333
Davis (P)	0.753	0.241	0.452	0.867	0.231	0.511	0.867	0.231	0.517	0.867	0.232	0.494	0.875	0.252	0.544
KIBA (D)	0.771	0.216	0.614	0.779	0.200	0.633	0.796	0.240	0.657	0.807	0.208	0.630	0.814	0.232	0.644
KIBA (P)	0.837	0.238	0.585	0.835	0.251	0.537	0.825	0.234	0.457	0.834	0.234	0.563	0.805	0.239	0.464

Note: Here, D means drugs and P means proteins.

Supplementary References

[1] Ahmad W, Simon E, Chithrananda S, Grand G, Ramsundar B. Chemberta-2: Towards chemical foundation models. arXiv preprint arXiv:2209.01712. 2022 nSep 5.

[2] Lin Z, Akin H, Rao R, Hie B, Zhu Z, Lu W, Smetanin N, Verkuil R, Kabeli O, Shmueli Y, dos Santos Costa A. Evolutionary-scale prediction of atomic-level protein structure with a language model. Science (American Association for the Advancement of Science), 379(6637), 1123–1130. https://doi.org/10.1126/science.ade2574.

[3] ESM Team. ESM Cambrian: Revealing the mysteries of proteins with unsupervised learning. Evolutionary Scale Website https://www. evolutionaryscale. ai/blog/esm-cambrian. 2024 Dec.

[4] Zhang H, Liu X, Cheng W, Wang T, Chen Y. Prediction of drug-target binding affinity based on deep learning models. Computers in biology and medicine. 2024 May 1;174:108435.

[5] Xu J, Ci L, Zhu B, Zhang G, Jiang L, Ye-Lehmann S, Long W. MMSG-DTA: A Multimodal, Multiscale Model Based on Sequence and Graph Modalities for Drug-Target Affinity Prediction. Journal of Chemical Information and Modeling 2025 65 (2), 981-996.

[6] Liu Z, Wang Y, Vaidya S, Ruehle F, Halverson J, Soljačić M, Hou TY, Tegmark M. Kan: Kolmogorov-arnold networks. arXiv preprint arXiv:2404.19756. 2024 Apr 30.

[7] Shah PM, Zhu H, Lu Z, Wang K, Tang J, Li M. DeepDTAGen: a multitask deep learning framework for drug-target affinity prediction and target-aware drugs generation. Nature Communications. 2025 May 30;16(1):5021.

[8] Kang Y, Zhuang H, Jiang Y, Li Z. MF-DTA: Predicting drug-target affinity with multi-modal feature fusion model. Journal of Biomedical Informatics. 2025 Oct 10:104926.
